# Association and dissociation of Na^+^ between bulk, cluster and micelle sites in aqueous sodium decanoate solutions elucidated by ^23^Na NMR relaxation experiments and quadrupolar relaxation modelling

**DOI:** 10.1039/d5sm01198a

**Published:** 2026-03-06

**Authors:** Pär Håkansson, Pau Mayorga Delgado, Anne Selent, Ritu Ghanghas, Ilari Ainasoja, Sanna Komulainen, Jiří Mareš, Perttu Lantto, Nønne L. Prisle, Ville-Veikko Telkki

**Affiliations:** a NMR Research Unit, University of Oulu P.O. Box 3000 FI-90014 Finland parhakansson22@gmail.com ville-veikko.telkki@oulu.fi; b Leiden Institute of Chemistry Einsteinweg 55 2333 CC Leiden Nederlands; c Department of Pharmaceutical Sciences, University of Vienna Vienna Austria; d Centre for Atmospheric Research, University of Oulu P.O. Box 4500 FI-90014 Finland; e Centre for Molecular Water Science CMWS, Deutsches Elektronen-Synchrotron DESY Notkestrasse 85 22607 Hamburg Germany; f Institute of Inorganic and Applied Chemistry, University of Hamburg Martin-Luther-King-Platz 6 20146 Hamburg Germany; g Centre for Material Analysis, University of Oulu P.O. Box 3000 FIN-90014 Finland

## Abstract

Counter-ion distribution in aqueous ionic surfactant solutions is a complex phenomenon, which is challenging to study experimentally. The degree of counter-ion binding to charged aggregates can significantly impact water activity. In atmospheric aerosols, which often include organic surfactants, such mechanisms may in turn strongly affect cloud droplet formation and earth's radiation balance. Here, we combine ^23^Na nuclear magnetic resonance (NMR) relaxation and diffusion experiments with advanced relaxation modelling for determining counter-ion dynamics and distribution in aqueous sodium decanoate solutions. Relaxation modelling of a complex system may require too many parameters to determine. Here, we assume, based our previous ^1^H NMR study, that below the critical micelle concentration (CMC), surfactants are monomers or form small clusters (about five decanoate ions), and above the CMC they form small clusters or larger micelles (about 48 decanoate ions). We propose two analytical relaxation models for the system. The number of adjustable parameters is reduced by molecular dynamics simulations. Our analysis indicates that below the CMC, the vast majority (about 97%) of Na^+^ counterions are unbound in the bulk, whereas above the CMC, a significant amount (36–58%) of Na^+^ ions are bound to micelles or clusters, greatly reducing the impact of both Na^+^ ions and surfactant aggregates on water activity. Also, Na^+^ ions associated with micelles undergo fast dynamics with sub nanosecond correlation times.

## Abstract 2

This study uses ^23^Na NMR and quadrupolar relaxation modelling to quantify Na^+^ partitioning between bulk water, aggregates, and micelles in decanoate solutions, revealing weak binding below the CMC and stronger association at higher concentration.

## Graphical abstract

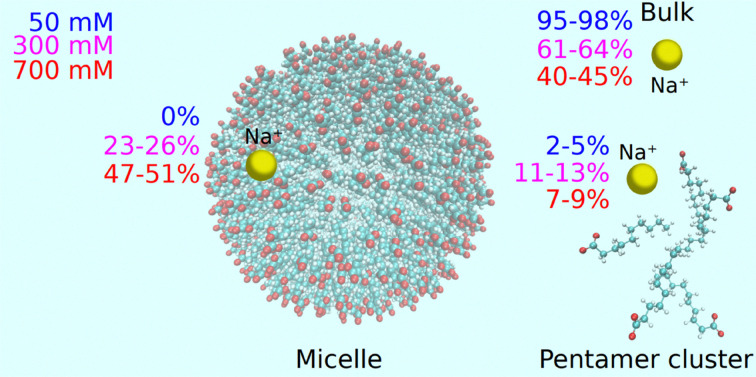

## 1. Introduction

Surface-active agents (surfactants) are typically organic compounds including a hydrophilic head group and a hydrophobic tail group.^1^ They tend to accumulate on the surface of a solution, lowering its surface tension. They are broadly used in science and technology as wetting agents, detergents, emulsifiers, foaming agents, dispersants, protein chemistry and food science.^2–5^ They are also common constituents of atmospheric aerosols and may affect cloud droplet formation and have aerosol-cloud-climate effects.^6–10^ Conventionally, it has been assumed that at lower concentrations surfactants exist as monomers in aqueous solutions, whereas at higher concentrations they tend to form aggregates such as micelles, vesicles, or lamellar phases.^11^ The aggregation may have a significant impact on the solution water activity. Ionic surfactants dissociate in aqueous solution to surfactant ions and counterions. Another important factor affecting water activity is counterion distribution, *i.e.*, the extent to which counterions are unbound in bulk water or bound to charged surfactant aggregates.

There are many thermodynamic models addressing counterion distribution and water activity.^12^ However, an inherent obstacle with these models is the need for an electrostatic continuous description of a system that at the molecular and aggregate level constitutes an oversimplification.^13^ Hence, to reach a sufficiently accurate quantitative estimates for a specific system, complementary studies are needed. Surfactant aggregation and phases in aqueous solutions have been studied with experimental techniques such as small-angle X-ray scattering (SAXS), laser and infrared spectroscopy, surface pressure measurements, scanning electron and atomic force microscopy, and neutron reflection.^14–20^ Lindman *et al.* determined the fraction of counterions bound to micelles in aqueous sodium decanoate and sodium *p*-octylbenzenesulfonate solutions based on self-diffusion measurements employing radioactive labelling and phenomenological models.^21,22^ Nuclear magnetic resonance (NMR) relaxation and diffusion measurements are powerful, non-invasive techniques to explore molecular organization and dynamics in heterogeneous systems,^23,24^ and the methods have also been broadly utilized to investigate surfactant aggregates.^21,25–27^

Recently, we combined experimental ^1^H relaxation and diffusion NMR studies with state-of-the-art relaxation modelling to investigate aggregation of surfactants in aqueous sodium decanoate solutions.^28^ Even though a conventional model assumes that surfactants exist as monomers below the critical micelle concentration (CMC) and above the CMC they form micelles,^17^ multiple studies have confirmed clustering below CMC (also called pre-micellar aggregation).^21,22,29–31^ Our studies implied that, below the CMC, clusters of decanoate ions included on average five surfactants.^28^ Furthermore, we showed that the size of micelles formed above the CMC is significantly smaller than predicted by conventional models. Based on thermodynamic modelling incorporating the size information, we estimated that, when atmospheric aerosols include these kinds of surfactants, the aggregation phenomena have a significant effect on the cloud droplet formation. This can lead to misrepresenting the aerosol cooling potential and bias predictions of climate models, if not considered appropriately.

^23^Na NMR relaxation is dominated by the strong (∼100 kHz) quadrupolar coupling,^25,32,33^ and typically the weaker relaxation mechanisms, such as dipolar and spin-rotation mechanisms,^24^ can be omitted in relaxation modelling to a good approximation. ^23^Na NMR relaxation studies have been utilized, *e.g.*, to study hexagonal phase and micelle size in the sodium dodecyl sulphate (SDS)/decanol/water system.^34,35^

In ^23^Na relaxation modelling, the motion of counterions along a micelle surface (i) has been found to constitute a dominating relaxation mechanism for larger micelles,^35^ providing geometry (radius) information. This mechanism has also been central in D_2_O and counterion relaxation in bilayer phases.^36,37^ Further propositions of mechanisms involve (ii) the movement of ^23^Na^+^ from micelle to water phase, (iii) micelle reorientation,^35^ (iv) the movement of surfactants to and from micelle and (v) the movement of water around ^23^Na^+^.^38^ To overcome a potentially large set of parameters, information can be incorporated from studies of other phases,^35^ or from molecular dynamics (MD) simulations providing the electric field gradient (EFG) fluctuations experienced by an atomic ion in ion-water systems.^39,40^

In this work, we report ^23^Na NMR relaxation and diffusion measurements of aqueous sodium decanoate samples with different concentrations (50, 300 and 700 mM) below and above the CMC (100 mM). Relaxation was measured at three temperatures (278, 283 and 295 K), diffusion at one (295 K). Detailed information about the dynamics and binding of Na^+^ counterions are extracted from the experimental data through comprehensive quadrupolar relaxation modelling. We utilize the information about cluster and micelle sizes from our previous ^1^H NMR relaxation and diffusion study of the same aqueous sodium decanoate system.^28^ Furthermore, the previous work provides us with the MD trajectories of the cluster and micelle systems as well as ^23^Na^+^ counterions, which are exploited to extract a part of the parametrization for the relaxation model. We note that the timescales of ion-dynamics determined by micelle and aggregate dynamics is longer than in the earlier studied inorganic ion-water systems,^39,40^ and therefore capturing the whole relaxation process from the few nanoseconds MD simulations is not possible. Hence, a parametrization of type (i) above is obtained from the simulation of the separate aggregate components and relevance of remaining potential mechanisms are discussed. In this way we obtain only a few adjustable parameters in a model to extract the fractions of unbound Na^+^ ions in bulk as well as Na^+^ ions bound to clusters and micelles. The synergy between parametrizations from MD and analytical relaxation models enables hypotheses to be tested that could not be explored with the larger set of unknown parameters otherwise encountered.^35^

## 2. Experimental

Sodium decanoate (98%, Sigma-Aldrich) was dissolved in D_2_O (99% D, Sigma-Aldrich). The solution was inserted into a 5 mm NMR tube, and the tube was sealed with a cap, Teflon tape and parafilm. Three samples with the concentrations of 50, 300 and 700 mM were prepared. The concentrations were selected to represent regions below the CMC (100 mM), between the CMC and critical vesicle concentration (CVC ≈ 550 mM) and above CVC, respectively.^28^

^23^Na NMR relaxation measurements were performed on Bruker Avance III 500 (11.7 T) spectrometer with the ^23^Na frequency of 132 MHz at three different temperatures (278, 283 and 295 K). *T*_1_ relaxation times were measured using the inversion recovery pulse sequence.^41^ The recovery time varied logarithmically from 0.001 to 0.5 s, the number of steps was 8 and the number of scans was 32. *T*_2_ relaxation times were measured using the Carr–Purcell–Meiboom–Gill (CPMG) sequence.^42^ The echo time was 0.49 ms, the number of echoes varied from 2 to 400 with 8 steps, and the number of scans was 8. The lengths of the 90° and 180° pulses were 20 and 40 µs.

^23^Na NMR diffusion measurements were performed on Bruker Avance III 600 (14.1 T) spectrometer with the ^23^Na frequency of 159 MHz at 295 K using the pulsed-field-gradient stimulated-echo (PGSTE) pulse sequence.^43^ The maximum gradient strength was 70 G cm^−1^, and the number of gradient steps was 16. The diffusion delay *Δ* (12–20 ms) and the length of the gradient pulse *δ* (1–3 ms) was optimized for each sample to obtain sufficient attenuation for the echo signal. The number of scans and dummy scans were 64 and 4, respectively. Examples of experimental data of *T*_1_ and *T*_2_ relaxation as well as diffusion experiments are shown in Fig. 2b–d. We note that, although quadrupolar nuclei often exhibit biexponential relaxation decay,^44^ the decay observed here is single-exponential.

**Fig. 1:**
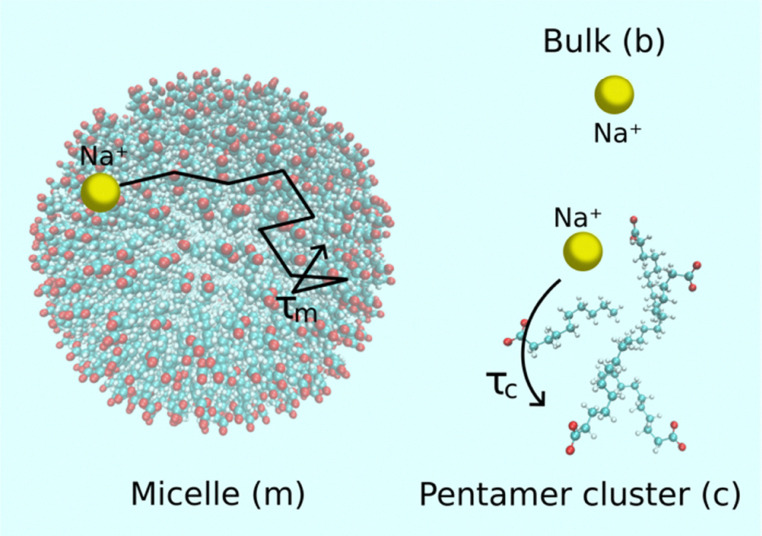
Three sites considered in the relaxation models: Na^+^ ions may be associated with micelles or smaller clusters including five surfactants, or they may be dissociated in bulk water. Corresponding mobility timescales of Na^+^ ions are *τ*_m_, *τ*_c_ and *τ*_b_, respectively.

**Fig. 2:**
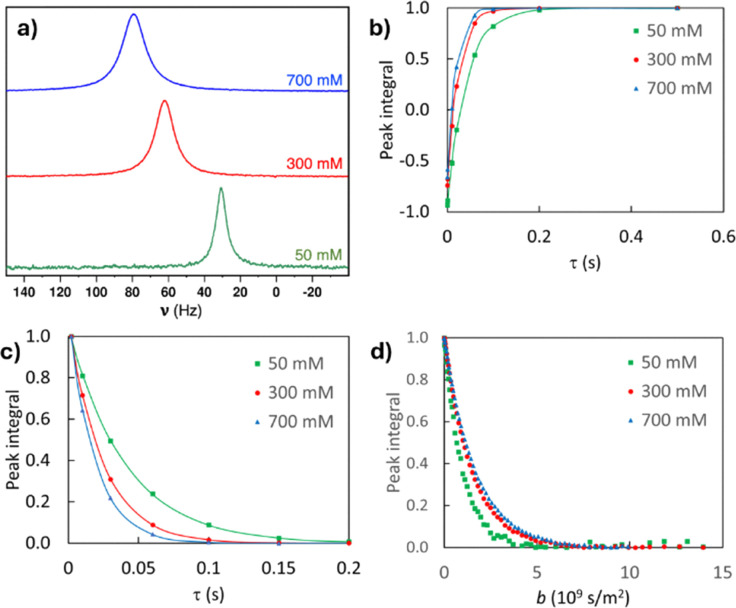
(a) ^23^Na spectra of the 50, 300 and 700 mM aqueous sodium decanoate samples measured in 11.7 T magnetic field at 298 K, represented on a frequency scale (*ν*). (b) ^23^Na inversion-recovery *T*_1_, (c) CPMG *T*_2_, and (d) PGSTE diffusion decay curves measured at 295 K.

To determine relaxation times and diffusion coefficients of dissociated Na^+^ ions in bulk water, we also prepared a low concentration sample including 10 mM NaCl dissolved in D_2_O, and performed the same variable temperature ^23^Na NMR relaxation time and diffusion experiments as for the sodium decanoate samples.

## 3. Relaxation modelling

A common starting point in ^23^Na relaxation modelling also followed in this work is the assumption of single exponential relaxation decay rates obeying the equations:^33^1
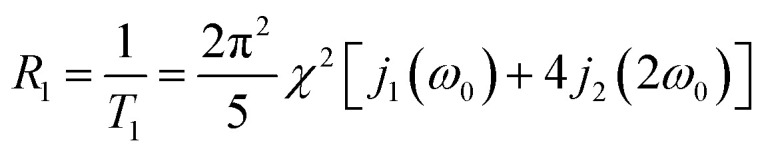
2
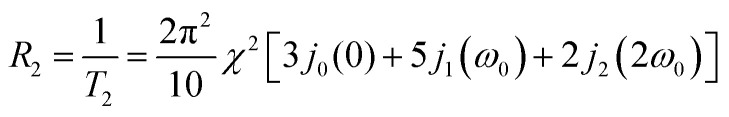
here, *R*_1_ and *R*_2_ are the longitudinal and transverse relaxation rates, respectively, *T*_1_ and *T*_2_ are the corresponding relaxation times, *χ* is the effective quadrupolar coupling, *j*_*n*_(*nω*_0_) (*n* = 0, 1 or 2) are the spectral densities, and *ω*_0_ is the ^23^Na angular resonance frequency. It is noted that *χ* is proportional to electric field gradient at the Na-ion,^24,33^ which has been experimentally determined for several surfactant sodium systems and found to be in the 100 kHz regime.^25,33,35^

### 3.1. Spectral density and translational diffusion model

To interpret experimental relaxation rates, we need a framework for the molecular dynamics leading to the spectral densities of eqn (1) and (2). In this work, we let the outcome of our previous ^1^H NMR study of the same aqueous sodium decanoate system to guide a plausible model for Na^+^ dynamics.^28^ As illustrated in Fig. 1, there are three Na-ion sites:

1. Above the CMC, Na^+^ ions can be in the proximity or within the micelle undergoing a motion at timescale *τ*_m_.

2. Over the whole surfactant concentration range, Na^+^ ions may follow a small (five surfactant) cluster at timescale *τ*_c_.

3. Dissociated Na^+^ ions can reside in bulk water

Because the exchange of Na^+^ ions between the three sites is fast in the time scale of ^23^Na NMR relaxation experiments, the spectral densities in eqn (1) and (2) are weighted averages of the spectral densities of Na^+^ ions associated with the surfactant aggregates and dissociated Na^+^ ions in the bulk:

3 *j*_*n*_(*nω*_0_) = *P*_a_*j*^a^_*n*_(*nω*_0_) + *P*_b_*j*^b^_*n*_(*nω*_0_)

Here, *P*_a_ and *P*_b_= 1 −*P*_a_ are the molar fractions of Na^+^ ions in aggregates (a), including both clusters (c) and micelles (m), and bulk (b), respectively. The parameter *j*^a^_*n*_ is the spectral density of the aggregates, which is the weighted average of the spectral densities of ions in micelles (*j*^m^_*n*_) and clusters (*j*^c^_*n*_):

4 *j*^a^_*n*_(*nω*_0_) = *X*_m_*j*^m^_*n*_(*nω*_0_) + *X*_c_*j*^c^_*n*_(*nω*_0_)

here, *X*_m_ and *X*_c_ = 1 − *X*_m_ are the relative fractions of Na^+^ ions in micelles and clusters, respectively. In eqn (3), *j*^b^_*n*_ is the spectral density of dissociated ions in bulk water. With the approximation of a single correlation time of each process, the spectral densities are given by5
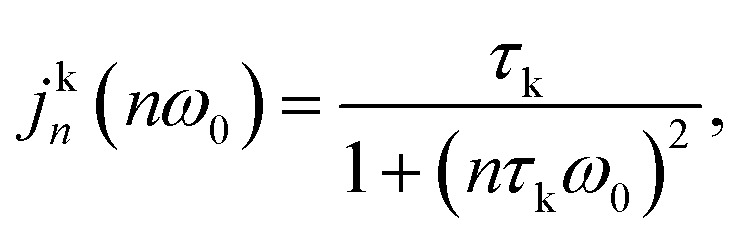
where k is c or m.

Due to the fast exchange of Na^+^ ions between the three sites, the observed effective translational diffusion coefficient *D*_eff_ is also a weighted average of the diffusion coefficients of Na^+^ ions associated with aggregates (*D*_a_) and dissociated Na^+^ ions in bulk (*D*_b_):

6 *D*_eff_ = *P*_a_*D*_a_ + *P*_b_*D*_b_.

here, *D*_a_ is a weighted average of translational diffusion coefficients of Na^+^ ions associated with micelles (*D*^tr^_m_) and smaller clusters (*D*_c_):

7 *D*_a_ = *X*_m_*D*^tr^_m_ + *X*_c_*D*_c_.

Relaxation times and diffusion coefficients of dissociated Na^+^ ions in bulk water were measured from the aqueous NaCl solutions. An underlying assumption in eqn (3) and (4) is that there is no significant correlation between the processes included in the separate spectral densities. To obtain a simple model for aggregate diffusion, we follow our previous work,^28^ where the unrestricted diffusion coefficient of aggregates is estimated by the Stokes–Einstein equation for spheres, and at higher concentrations the diffusion is slightly reduced given by geometrical obstruction, *i.e.*, interaction of aggregates with each other.^45,46^ This type of decomposition is frequently used in quadrupolar and other relaxation/diffusion studies.^24,25,34,38^ The relative fraction of surfactants in micelles is

8 *X*_m_ = (*C* − CMC)/*C*

where *C* and CMC are the total concentration and critical micelle concentration of surfactants.^22^ The fraction of Na^+^ ions associated to micelles and clusters is assumed to be directly proportional to the fraction of surfactant ions in micelles and clusters, respectively. Hence, the actions are *X*_m_*P*_a_ and *X*_c_*P*_a_, respectively.

### 3.2. Molecular dynamics simulations

MD trajectories were computed with the AMBER program package,^47^ and are presented in detail in our previous work.^28^ Here, we analyse simulations of 66 surfactant micelles and pentamer clusters at the temperatures of 278, 283 and 295 K. These micelle and cluster sizes were used to analyse surfactant dynamics in our previous work based on the three-site model illustrated in Fig. 1, although different sizes were explored as well.^28^

The pentamer clusters were computed with a quantum mechanics/molecular mechanics (QM/MM) method. In these cases, the set of surfactants was treated with the QM method at every time step, providing the forces that enter the simulations, whereas the solvent water molecules and sodium ions were described classically. The micelle simulations with 66 surfactants were simulated with the classical MM.^28^

Common for all simulations is the flexible single point charge water force field (SPC/Fw),^48^ and the steps conducted to reach the equilibrated temperatures. The setup of each system involves the initial energy minimization of the complete system (water, surfactant ions, and sodium ions) consisting of 6000 steps, with alternating steepest descent and conjugate gradient algorithms. All systems underwent first a 30 ps constant volume temperature equilibration to reach the sought temperature in contact with the Langevin thermostat.^49^ Subsequently, equilibration was performed with a constant number of particles as well as constant pressure and temperature (NPT), with the target temperature and volume altered to reach on average the 1 bar pressure target with 0.3–6 ns timespans using also the Berendsen barostat. The production runs were carried out with NVT ensemble. The particle-mesh Ewald method^47^ was used for the long-range electrostatic interactions, with a cutoff at 9 Å. The simulations were performed in a cubic box with periodic boundary conditions, and the typical box dimension varied between 38 and 68 Å.^28^ The surfactants in micelle simulation have the GAFF constant partial charge force field.

In the simulations for the pentamer cluster, the QM region was embedded in the explicit SPC/Fw waters mentioned above. The QM region was modelled with the parameter model 3 (PM3) Hamiltonian,^50^ a semiempirical QM method.

From the MD simulations, we computed the average residence time of Na^+^ ions within 35 Å from the centre of mass of the micelle. This was achieved by tracking those ions staying within that region for 100 ps or more, which we call an event. Thus, the average residence time is obtained as the mean of the time for these events. The averaged residence time over all the ions includes those sodium ions that stayed within the micelle for the whole trajectory.

To support the simplification of classical MD force field with static partial charges,^40^ a model for dynamics where all classical MD is omitted and replaced with a parametrization is tested separately.

## 4. Results and discussion

### 4.1. Experimental results

^23^Na NMR spectra of the 50, 300 and 700 mM aqueous sodium decanoate samples measured at 298 K are shown in Fig. 2a. The spectra do not show quadrupolar splitting, indicating that on the NMR timescale, Na^+^ experiences an isotropic environment. Isotropic tumbling of Na^+^ ions is natural in the 50 mM sample, as its concentration is below the CMC (100 mM), and Na^+^ ions are expected to be predominantly unbound in the water solvent. As illustrated in Fig. 1, in the higher concentration samples, some Na^+^ ions are expected to be associated with the small clusters and larger micelles. The lack of quadrupolar splitting indicates that the diffusion of Na^+^ ions within the clusters and micelles as well as their exchange between the three sites is fast on the NMR time scale. The resonance frequency (chemical shift) of the ^23^Na signal increases with concentration, most likely due to Na^+^ binding to the surfactant headgroups.^51^

^23^Na *R*_1_ and *R*_2_ relaxation rates of the aqueous sodium decanoate samples increase with increasing concentration because of slower motion of Na^+^ ions due to the association with the surfactant aggregates (Fig. 4a and b). The rates also increase with decreasing temperature due to slower dynamics. The relaxation rate varies between 24 and 86 s^−1^ (*T*_1_ and *T*_2_ vary between 12 and 42 ms). *T*_1_ deviates only 3–5% from *T*_2_, indicating relatively fast molecular processes with correlation times less that *ω*_0_^−1^ ≈ 1.2 ns close to extreme narrowing region.^24^

**Fig. 3:**
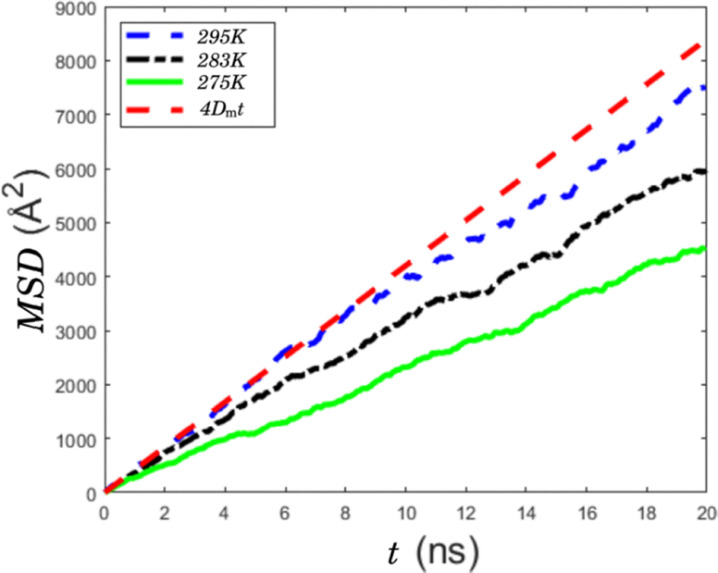
Mean square displacement (MSD) of Na^+^ in a reference frame fixed at the micelle center of mass. The red dashed line is the fit of equation MSD = 4*D*^s^_m_*t* with the initial slope at 295 K.

**Fig. 4:**
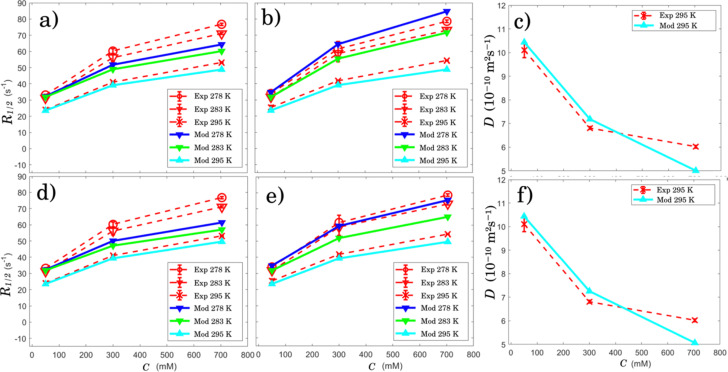
Panels (a), (b) and (c) show a fit of model 1 (Mod) with the experimental (Exp) ^23^Na NMR for the longitudinal (*R*_1_) and transversal relaxation rates (*R*_2_) as well diffusion coefficients of the aqueous sodium decanoate samples as a function of surfactant concentration, respectively. Model 1 considers free ions, small aggregates of five surfactants and larger micelles of radius 27 Å. Panels (d), (e) and (f) show the same kind of fits as with the model 1 but for the model 2 (Mod) with the experimental (Exp) ^23^Na NMR. The order of the plots is the same for both models for better comparison. Ion diffusion occurs at an adjustable radius *R*_m_ in model 2.

^23^Na relaxation rates of the 10 mM aqueous NaCl sample (*R*_1_ = 22.1 and 30.4 s^−1^, *R*_2_ = 22.8 and 31.0 s^−1^ at 295 and 283 K, respectively), representing the relaxation of dissociated Na^+^ ions, are close to the values for 50 mM sodium decanoate, suggesting that the surfactants have only a minor influence on ^23^Na relaxation below CMC, whereas above the CMC the surfactants increase the relaxation rates significantly. ^23^Na translational diffusion coefficient of 10 mM aqueous NaCl solution at 295 K is 10.7 10^−10^ m^2^ s^−1^, which is very similar to the Na^+^ diffusion in the 50 mM decanoate solution, implying that the Na diffuses independently from the monomeric decanoate.

### 4.2. Diffusion parameterization from MD simulation

The key motional process in ^23^Na quadrupolar relaxation above the CMC is the motion of Na^+^ in the charged micelle environment. This process is commonly parametrized with a lateral or surface diffusion constant that by definition of the diffusion process may involve shorter times trapped in micelle pockets.^25,34,52^ For a system where aggregate reorientation is negligible (such as for a large or fixated aggregate), this becomes a true surface diffusion constant.^52^ However, for the micellar system considered here, the process is a mixture of micelle reorientation and surface diffusion, the latter denoted with coefficient *D*^s^_m_. The surface diffusion of the Na^+^ on the micelle is estimated from the MD simulations, where the mean square displacement (MSD) of the ions in proximity to the micelle is recorded in a reference frame where center of mass of the micelle is fixed. This gives the temperature dependent MSD as shown in Fig. 3, where the surface diffusion is estimated from the initial slope. The focus is on the initial slope since at longer times the ion may leave or follow a complete micelle rotation. The estimates are given in Table 1. The estimated *D*^s^_m_ value at 295 K is 10.610^−10^ m^2^ s^−1^, which is similar to the surface diffusion coefficient estimated for single ion surfactants in hexagonal phase at 298 K (4.810^−10^ m^2^ s^−1^).^32,34^ The correlation times *τ*_m_ calculated using the *D*^s^_m_ assuming diffusion on an interface approximated by a sphere.^53^ The correlation times (Table 1) are similar and slightly shorter than the MD estimate of micelle reorientation correlation times.^28^

**Table 1:** Estimates of surface diffusion coefficients *D*^s^_m_ and correlation times *τ*_m_ from the MD mean square displacement of Na^+^ ions in proximity of micelles. Correlation times are calculated by *τ*_m_ = *R*^2^_m_/6*D*^s^_m_, where *R*_m_ is the micelle radius (27.0 Å)^28^

*T*/K	*D* ^s^_m_/10^−10^ m^2^ s^−1^	*τ*_m_/ns
275	7.4	1.6
283	9.1	1.3
295	10.6	1.1

We note that conclusions from the MD micelle simulations need caution since fixed charges are used, which is a possible oversimplification. Our *D*^s^_m_ estimate is of the same order of magnitude as the literature observation (a factor of two larger, see above), making it worthwhile to explore what the temperature dependence of *D*^s^_m_ give in relaxation model. The *τ*_m_ values calculated using *D*^s^_m_ and micelle radius are much longer than the values given by the model fits (see below). The deviation may have several sources, where a plausible reason for the faster motion obtained in the model fits is the overall rotation of micelle.

According to the MD simulations, the Na^+^ residence time in micelles is long, with a mean exceeding 10 ns for a 35 Å boundary radius. In Section 3.2 we describe how these residence times are obtained. In the relaxation models, we have omitted including this residence time and discussed afterwards what consequence it may have.

The correlation time *τ*_c_ for the pentamer cluster (radius *ca.* 6.4 Å) were estimated in the same way as for micelles, and the resulting values are 0.42, 0.34 and 0.29 ns at 275, 283 and 295 K, respectively.

### 4.3. Model 1

Next, we perform a global fit eqn (1), (2) and (6) with experimental *R*_1_, *R*_2_ and *D* values at three different concentrations and temperatures (*D* measured only at one temperature) using a Markov chain monte carlo (MCMC) procedure.^28,54^ Example code for model 1 and 2 is provided in Zenodo.^55^ In model 1, we assume that *τ*_m_^−1^ can be represented by the Arrhenius equation^24^9
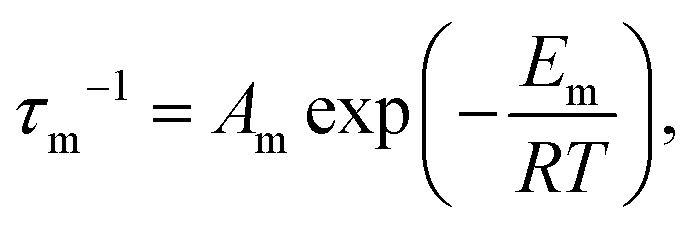
providing temperature dependence and two adjustable parameters, the pre-exponential factor *A*_m_ and the activation energy *E*_m_. We do not use the *τ*_m_ values extracted from the MD simulations (Table 1), because the fixed partial charges may be an oversimplification when estimating ion dynamics. Additional adjustable parameters in model 1 are the molar fractions of ions in aggregates *P*_a_ (assumed to be independent of temperature) for each concentration and the quadrupolar coupling, which is constrained to the narrow interval 99–119 kHz based on previous observations.^32,34,37,56^ This makes up a total of 6 adjustable parameters. The molar fraction *X*_m_ is fixed, given by eqn (8), and the correlation times for pentamer cluster (*τ*_c_) are given by the MD simulations (see previous section).

Eqn (1), (2) and (6), based on model 1, fit with the experimental data rather well, although *R*_1_ values are systematically slightly underestimated (see Fig. 4a–c). The fit results in the correlation times *τ*_m_ = 0.59 ± 0.04, 0.46 ± 0.02 and 0.32 ± 0.01 ns at 275, 283 and 295 K, respectively, activation energy *E*_m_ = 20.9 ± 0.5 kJ mol^−1^, pre-exponential factor *A*_m_ = (1.6 ± 0.3) × 10^13^ s^−1^ and quadrupolar coupling constant 117.00 ± 0.01 kHz. The site fractions are listed in Table 2. The small error estimate for quadrupolar coupling is due to its quadratic dependenece (eqn (1) and (2)) together with the constraints discussed and the small number of parameters used in the model.

**Table 2:** Molar fractions of ^23^Na^+^ ions at micelle, pentamer-cluster, and the bulk sites, given with 99% confidence intervals from MCMC sampling of model 1

Surfactant concentration (mM)	Micelle *X*_m_*P*_a_	Pentamer-cluster *X*_c_*P*_a_	Bulk *P*_b_
50	0.0	0.03 [0.02, 0.04]	0.97 [0.96, 0.98]
300	0.25 [0.25, 0.26]	0.13 [0.12, 0.13]	0.62 [0.61, 0.63]
700	0.50 [0.48, 0.51]	0.08 [0.08, 0.09]	0.41 [0.40, 0.42]

The correlation times *τ*_m_ provided by model 1 (0.3–0.6 ns) are much shorter than those estimated from the MD simulations (Table 1, 1.1–1.6 ns). This may be a consequence of oversimplification due to the fixed partial charges in the MD simulations. Furthermore, the MD simulations analysis assumes that the main mobility happens at the geometric radius of the micelle, while the model 1 does not include any assumption of the position of Na^+^ ions with respect to the centre of mass of micelles.

### 4.4. Model 2

In model 2, surface diffusion coefficient *D*^s^_m_ is taken from the MD simulations (Table 1). Correlation time *τ*_m_ is calculated by equation *R*^2^_m_/6*D*^s^_m_, similar to Table 1, but the actual radius *R*_m_, where the dominating translation of ions occur at micelle, is one adjustable parameter, being the same for all concentrations and temperatures. Hence, the total number of adjustable parameters is 5, reduced by one compared to model 1. Model 2 allows us to also explore how well the MD temperature dependence works. The fixed parameters and constraints are the same as in model 1.

MCMC analysis gives surface diffusion radius of *R*_m_ = 14.5 ± 0.3 Å, leading to correlation times of *τ*_m_ = 0.47 ± 0.02, 0.38 ± 0.02 and 0.33 ± 0.01 ns. The quadrupolar interaction becomes 117.00 ± 0.01 kHz and the site fractions are listed in Table 3. The mean MCMC parameter estimates for model 2 have observables given in Fig. 4 d, e and f.

**Table 3:** Molar fractions of ^23^Na^+^ ions at micelle, pentamer cluster, and the bulk sites, given with 99% confidence intervals from MCMC sampling of model 2

Surfactant concentration (mM)	Micelle *X*_m_*P*_a_	Pentamer-cluster *X*_c_*P*_a_	Bulk *P*_b_
50	0.0	0.03[0.019,0.05]	0.97 [0.95, 0.98]
300	0.24 [0.23, 0.26]	0.12 [0.11, 0.13]	0.63 [0.62, 0.64]
700	0.49 [0.47, 0.50]	0.08 [0.07, 0.09]	0.43 [0.41, 0.45]

### 4.5. Comparison of models 1 and 2

Within the 99% confidence interval, model 2 leads to molar fractions equal to model 1 (Tables 2 and 3). Below the CMC, nearly all Na^+^ counterions (95–98%) remain unbound in the bulk solution. Above the CMC, a substantial fraction becomes associated with micelles or clusters, increasing from 36–39% at 300 mM to 55–60% at 700 mM. Using self-diffusion measurements with radioactive labelling and a phenomenological model, Lindman and Brun^22^ reported a 60% degree of counterion binding at the CMC in aqueous sodium octanoate – somewhat higher than our value at 300 mM (36–39%). This discrepancy may partly reflect the longer hydrocarbon chain of decanoate relative to octanoate. It may also arise from differences in the underlying models: the phenomenological framework of Lindman and Brun considers only monomers and micelles, whereas our analysis incorporates the coexistence of micelles and small clusters, based on the microscopic information provided by our ^1^H relaxation and diffusion data together with relaxation modelling and MD simulations.^28^ We note that combining relaxation and diffusion measurements has the potential to yield substantially more detailed information about aggregate structure than diffusion data alone.

Model 2 describes *R*_2_ values better than model 1 (Fig. 4b and e), and it leads to slightly shorter *τ*_m_ values at lower temperatures. Because *R*_m_ (14.5 Å) obtained by model 2 is significantly smaller than the radius of a micelle (27 Å), the model implies that Na^+^ ions are predominantly trapped inside the micelle, and the MD simulations show that they stay there for a long time (exceeding 10 ns). It is possible to replace *τ*_m_ by *τ*_eff_eqn (5), where *τ*_eff_^−1^ = *τ*_m_^−1^ + *τ*_res_^−1^ takes also into account of the residence time *τ*_res_ of Na^+^ ion in micelle. However, as the residence time *τ*_res_ is orders of magnitude longer than the correlation time *τ*_m_, the latter would still be the dominating factor. Thus, considering ion sites in Fig. 1, there are ions inside and outside the micelle, but the residence time *τ*_res_ is too long to contribute in any larger degree to the quadrupolar relaxation. Previous work on Na^+^ relaxation in long range ordered surfactant phases did not indicate the need for exceptionally long residence time within aggregates;^34^ instead, it indicated correlation times in nanosecond range. Future explorations could shed light on the role of the force field in altering the Na^+^ residence time or changing local mobility of water or surfactants.

Models 1 and 2 agree with the experimental observations best below CMC at 100 mM (see Fig. 4). In that region, the sample is assumed to include only unbound bulk ions and smaller cluster with 6.4 Å radius. This is the simplest system that is also consistent with our previous study.^28^ However, a more complex model with broader aggregate size distribution would also be consistent with our results.

Both models show a deviation at higher concentrations for diffusion coefficients *D*. This may be a consequence of neglecting the existence of aggregates with various sizes and even vesicles above the CVC indicated in our previous study.^28^ Counterion binding to the larger aggregates (vesicles) may reduce observed, exchange-averaged diffusion coefficient. Another possible venue for further work is to consider diffusion obstruction for charged micelles. Obstruction model in this work accounts for geometric size.^45,46^ However, the presence of larger aggregates could introduce additional diffusion obstruction, which is not accounted for in the model used in this work.

Both models show similar deviations in *R*_1_, suggesting that single correlation time model is not completely sufficient. However, introducing a sum of spectral densities give more parameters without necessarily increased physical insight.^54^

We did not test relaxation modelling using a standard surfactant monomer-micelle-model, without clusters. Hence, it is yet not explored if monomer would give a worse or acceptable agreement. However, it would lead to incorrect proportions of unbound and bound Na^+^ ions, because, according to our ^1^H studies,^28^ the monomer/micelle model is incorrect.

In the ^23^Na relaxation modelling, we assumed that the average decanoate surfactant cluster size (5) remains constant across the studied temperature range. While temperature-dependent variations in cluster size are certainly possible, incorporating such effects would have introduced additional complexity into the model, so we chose not to include them.

## 5. Conclusions

We combined ^23^Na NMR spectroscopy, relaxation and diffusion experiments with relaxation modelling to understand dynamics and association/dissociation of Na^+^ ions in aqueous sodium decanoate solutions as model systems of atmospheric aerosol droplets. The lack of quadrupolar splitting in the spectra revealed that, on the NMR time scale, Na^+^ ions experience an isotropic environment due to the relatively fast diffusion of Na^+^ ions within the clusters and micelles as well as their exchange. In the modelling, based on our previous ^1^H NMR study of the same systems,^28^ we considered three sites, including dissociated Na^+^ ions in bulk water, as well as Na^+^ ions associated with small pentamer clusters and larger micelles. The correlation times for Na^+^ ions in pentamer clusters (*τ*_c_) are given by the MD simulations. In model 1, correlation times of Na^+^ ions in micelles *τ*_m_ were represented by the Arrhenius equation with the pre-exponential factor and activation energy being adjustable parameters, while in model 2 *τ*_m_ was calculated from the surface diffusion coefficient obtained from the MD simulations, with the surface diffusion radius being an adjustable parameter. The models give similar, sub-nanosecond *τ*_m_ values as well as equal fractions of ions in the three sites within the 99% confidence interval, despite their differing assumptions about slower dynamics, enhancing the reliability of the association analysis. It is found that the partition of previous work^28^ is sufficient and does not need refinements to also be used in the present ^23^Na study. With the increased parameter space without MD simulations would, if possible, drastically weaken such confirmation. The models predict that, at the lowest (50 mM) concentration, which is below the CMC (100 mM), 97% of the Na^+^ ions are unbound in bulk water and only 3% associated with the small clusters. However, at the highest (700 mM) concentration, the majority (58%) of the Na^+^ ions are bound to either clusters (8%) or micelles (50%). This is valuable information for better understanding the impact of dissolved sodium decanoate and other self-aggregating ionic surfactants on water activity as a key parameter in cloud droplet formation and climate effects of atmospheric aerosols.^28^ Sodium ions bound to clusters or micelles will have significantly lower ability to reduce water activity^57^ and promote water condensation to small droplets.^58^ The work also addresses potential issues related to the ion dynamics obtained from the MD simulations due to a simple force field used. Better understanding the connection between quantitative details and MD force fields may help in model building^59^ and understanding of the physical chemistry of this and more complex surfactant systems. Because our parallel study provides quantitative information on decanoate aggregation from ^1^H NMR relaxation and diffusion measurements of the same aqueous sodium decanoate system,^28^ and the present work offers complementary insight into Na^+^ counterion binding from ^23^Na NMR, the two studies together deliver a comprehensive picture of the aggregation phenomena. This combined information can be used for more accurate estimation of water activity, aerosol hygroscopic growth, cloud droplet formation, and their influence on aerosol cooling potential, was done through thermodynamic modelling described in ref. 28.

## Author contributions

Pär Håkansson: corresponding author, planning and performing the relaxation modelling with MD simulations, writing the first version of the manuscript. Pau Mayorga Delgado: performing MD simulations, computing the residence time of Na^+^ within micelles, conceptualization of Fig. 1 and revision of the manuscript. Anne Selent, Ritu Ghanghas, Ilari Ainasoja, Sanna Komulainen: sample preparation, performing ^23^Na relaxation and diffusion experiments, analyzing experimental data. Jiří Mareš and Perttu Lantto: supervision. Nønne L. Prisle: original idea of the systems to study and the purpose of it. Ville-Veikko Telkki: supervision, rewriting the first draft, helping with conceptualization and interpretation. All the authors contributed to the final version of the manuscript.

## Conflicts of interest

There are no conflicts to declare.

## Data Availability

All essential data of the research is given in the manuscript text, figures and tables. Example code for model 1 and 2 is provided in Zenodo: Parcode, Zenodo, 2026, preprint, DOI: https://doi.org/10.5281/zenodo.18211877.
